# Lung Carcinoids in Adolescents and Young Adults (AYAs): A Still Overlooked Clinical Entity

**DOI:** 10.3390/curroncol32080458

**Published:** 2025-08-14

**Authors:** Alice Laffi, Laura Pala, Chiara Catania, Marzia Locatelli, Priscilla Cascetta, Emilia Cocorocchio, Giovanni Luca Ceresoli, Daniele Laszlo, Flaminia Facella, Emily Governini, Marzia Bendoni, Giuseppe Pelosi, Fabio Conforti, Tommaso Martino De Pas

**Affiliations:** 1 Division of Medical Oncology, Humanitas Gavazzeni, 24125 Bergamo, Italy; laura.pala@gavazzeni.it (L.P.); chiara.catania@gavazzeni.it (C.C.); marzia.locatelli@gavazzeni.it (M.L.); priscilla.cascetta@gavazzeni.it (P.C.); emilia.cocorocchio@gavazzeni.it (E.C.); giovanni.ceresoli@gavazzeni.it (G.L.C.); daniele.laszlo@gavazzeni.it (D.L.); flaminia.facella@gavazzeni.it (F.F.); emily.governini@humanitas.it (E.G.); marzia.bendoni@humanitas.it (M.B.); fabio.conforti@hunimed.eu (F.C.); tommaso.depas@gavazzeni.it (T.M.D.P.); 2 Tumor Microenviroment Unit, IRCCS Humanitas Research Hospital, 20089 Milan, Italy; 3 Department of Biomedical Sciences, Humanitas University, 20072 Milan, Italy; 4 Department of Oncology and Hemato-Oncology, University of Milan, 20122 Milan, Italy; giuseppe.pelosi@unimi.it

**Keywords:** neuroendocrine tumors, NETs, lung carcinoids, AYA

## Abstract

Pulmonary carcinoids (PCs) are rare neoplasms involving typical and atypical carcinoids (TCs and ACs), defined histologically by absent or focal necrosis and mitotic counts (<2/mm^2^ vs. 2–10/mm^2^), respectively. Although uncommon overall, TCs and ACs represent the most frequent non-hematologic malignancies in the pediatric population. However, significantly less is known about PC in AYAs, a population often overlooked or analyzed within pediatric or adult cohorts. In this critical review, we analyzed existing literature on PCs in the AYA population using a question-and-answer format, emphasizing the substantial gap in current knowledge in this field and the urgent unmet clinical need for future scientific proposals. First, we analyzed epidemiology and the data availability about the association between PCs in AYA patients and genetic syndromes that typically reach the maximal diagnostic incidence within this age group. We then reviewed the available literature about the pathologic characteristics, clinical presentation, and treatment strategies for localized and metastatic disease in PC AYA patients. According to our findings, a significant lack of age-specific evidence and the need for international collaboration and prospective, AYA-focused clinical studies were underscored. Advancing research in this area is essential to improve understanding and develop tailored, evidence-based therapeutic approaches for this peculiar population.

## Simple Summary

Pulmonary neuroendocrine tumors (NETs) represent a rare and heterogeneous category of neoplasms. Although uncommon, they are among the most common lung tumors in adolescents and young adults (AYAs), a population often neglected or included within broader pediatric or adult cohorts. Nevertheless, a comprehensive understanding of AYA oncology and effective management of these conditions in this peculiar population remains an unmet clinical need, due to both healthcare-related and psychosocial challenges that characterize AYA patients. This critical review aims to highlight current knowledge of pulmonary NETs in this unique category and identify areas that still require further investigation.

## 1. Introduction

Well-differentiated neuroendocrine tumors (NETs) of the lung are a heterogeneous category of rare neoplasms, accounting for up to 2% of all primary pulmonary cancers [1]. Although their overall incidence is low, lung NETs constitute one of the most frequent thoracic malignancies in younger individuals. Within this spectrum, adolescent and young adults (AYAs)—defined by the U.S. National Cancer Institute as individuals aged between 15 and 39 years—represent one of the most neglected subgroups in oncologic research and clinical trials [2].

Despite data about different clinical and psychosocial features of AYA patients, most evidence regarding diagnosis, treatments, and outcomes in lung NETs is derived from pediatric or adult cohorts. For this instance, we designed this critical review with the aim of highlighting the paucity of data and the unmet clinical needs of AYA patients with pulmonary carcinoids. By raising awareness within the scientific community, we aim to encourage the development of age-tailored diagnostic and therapeutic strategies and foster collaborative research initiatives that could improve the knowledge of lung NETs in AYA patients and the outcomes for this overlooked population.

## 2. Epidemiology

### 2.1. What We Know

The incidence is around 0.2–2/100,000 population/year in the US and Europe, and they account for 1–2% of all lung cancers [1]. According to the international guidelines, lung neuroendocrine tumors (NETs) are divided into typical and atypical carcinoids (TCs and ACs) based on the mitosis count (<2/mm^2^ vs. 2–10/mm^2^) and the presence of necrosis (absent vs. focal). According to histological characteristics, TCs occur earlier compared to ACs, with a younger median age at diagnosis compared to the other cancers [2,3]. This trend is especially pronounced in the case of genetic syndromes, the most common being multiple endocrine neoplasia type 1 (MEN-1). In this regard, adolescent and young adult (AYA) patients, a peculiar and often underappreciated population aged between 15 and 39 years (National Cancer Institute. Adolescents and young adults with cancer [Available from: https://www.cancer.gov/types/aya, accessed on 13 August 2025], are notably affected by pulmonary carcinoids (PCs) [2,4,5,6,7,8,9,10]): up 80–85% of primary lung neoplasms in children are PCs that account 16.5% of all pediatric malignancies [11]. A similar experience at the M. D. Anderson Cancer Center was reported by Broaddus RR et al., where lungs are the most common origin for pediatric and adolescent NETs [12].

### 2.2. What We Do Not Know

Given the limited available data and the paucity of studies addressing PCs in AYA patients, the incidence and the clinical impact of PCs within this population remain, to our knowledge, unknown. The studies presented span different periods, during which various classifications have been introduced, alongside significant advancements in the understanding of NETs. For these reasons, national or international databases—comparable to those established for more common cancers—should also be developed for rare diseases within specific subcategories of the population, raising awareness across the entire scientific community, including pediatricians, endocrinologists, thoracic surgeons, and oncologists, regarding the importance of contributing to these efforts.

## 3. Clinical Presentation

### 3.1. What We Know

Having outlined the epidemiology of pulmonary carcinoids in the AYA population, it is important to examine their clinical presentation, as early recognition of symptoms can significantly impact diagnostic and therapeutic strategies.

The study by Abele M. et al. [13] on lung cancers in Germany reported a predominance of PCs in the pediatric and AYA population, with a higher prevalence in males. According to earlier studies [2,14], PCs in patients under 17 years old are mostly endobronchial [15]. Consequently, the most commonly reported symptoms include cough, wheezing, hemoptysis, or pneumonia [11,12,15].

As observed in other AYA oncologic diseases [16], symptoms often begin during adolescence and are typically present for over a year before diagnosis [17]. Despite this delay, the largest available cohort shows that the disease is most often in a localized stage at diagnosis in AYA patients [12,15].

The most commonly used tools for diagnosis and staging include bronchoscopy, chest CT scan, ^68^Gallium (^68^Ga)-DOTA-peptide PET imaging, and, less frequently, ^18^Fluorodeoxyglucose (FDG) PET scans [18,19]. Such as in adult PCs, functional imaging results can vary widely between patients. Some tumors show strong uptake on ^68^Ga scans due to somatostatin receptor expression, while others may be ^68^Ga-negative but show uptake on FDG PET scans.

Although rare, PCs in AYA patients can be associated with a clinical syndrome that may present before tumor detection. For instance, Vaca R. et al. [20] reported the case of a female patient who initially presented with signs of ectopic Cushing’s syndrome (ECS). Following the endocrinologic evaluation, a chest CT scan revealed a lung nodule. Surgical resection confirmed an AC, and the ECS symptoms resolved postoperatively.

Lastly, in some cases, PCs are linked to genetic syndromes, commonly MEN-1, an autosomal dominant inherited condition [21], and rarely MEN-4. In patients with MEN-1 undergoing thoracic imaging, the estimated prevalence of PCs is around 13%, regardless of age. In the study by De Laat JM et al. [22], 16 out of 323 MEN-1 patients with PCs were identified, including 6 AYA patients. Among these, only one was a current smoker, and most of them presented with a localized disease at diagnosis.

### 3.2. What We Do Not Know

In the case of AYA patients with nonspecific symptoms suggestive of a lung neoplasm, the potential radiological impact during the diagnostic phase often raises ethical concerns, which may contribute to delays in diagnosis. A chest X-ray is typically inconclusive unless atelectasis is present, which instead strongly justifies further imaging through a CT scan. The same challenge applies to patients presenting with clinical syndromes, where the diagnosis requires specialized expertise from endocrinologists and close collaboration between different specialists. Therefore, establishing an international registry to collect cases of AYA patients with PCs could help assess the scope of the issue and raise awareness among healthcare providers who may face the AYA population.

In the absence of specific diagnostic and therapeutic care pathways for AYA patients, it seems reasonable to manage those with a histologic diagnosis of PC similarly to adult PC patients. This would include completing staging with ^68^Ga PET, and if negative, proceeding with FDG PET before any therapeutic approach.

However, at present, some AYA PC patients undergo surgery without completing the staging process or a multidisciplinary evaluation, and effective cooperation between specialists remains something that still needs to be fully implemented.

Moreover, in this context, the scientific community also lacks knowledge about the impact that a diagnosis of PCs (and MEN-1 syndrome) may have on the reproductive and professional life of AYA patients. An AYA patient, particularly between 18 (or 21 for some countries) and 39 years of age, is an individual transitioning into professional life, reaching peak reproductive potential, or having recently started a family, factors that may significantly influence the psychosocial and clinical implications.

## 4. Pathology

### 4.1. What We Know

Currently, there are no studies specifically addressing the pathological characteristics of PCs in the AYA population. The differential diagnosis between TCs and ACs requires a surgical specimen in all patients, irrespective of age [1]. Nevertheless, it is commonly observed that TCs tend to develop in younger patients compared to ACs. Unfortunately, in most existing studies, AYAs have been included with the broader adult populations [23,24]. Potter SL et al. reported a higher incidence of TCs in the pediatric and AYA populations compared with the adults [25]. However, adding further complexity to the situation, several studies including AYA and pediatric patients presented data with the risk of confounding; some papers report only AC cases [26,27], other focused only on AYA patients with TCs [15,17,19,20,28,29], while other include both the histotypes or provide no specific classification (not otherwise specified—NOS) [2,18,22]. Also, the immunohistochemical (IHC) demonstration of PCs passes through specific neuroendocrine markers such as chromogranin A, synaptophysin, and/or CD56, cytokeratins, and thyroid transcription factor-1 (TTF-1). From the non-small cell lung cancer literature, TTF-1, a well-known biomarker for lung neoplasms, may be negative in never-smokers [30], and similar findings have been reported about PCs [31]. La Salvia et al. observed a non-significant trend in TTF-1 expression analyzed by age [32]. More notably, Papaxoinis G et al. identified three different clinical clusters, each associated with unique pathological patterns based on TTF-1 and OTP expression. In this paper, patients within the TTF-1 (−)/OTP (+) cluster were significantly younger than those in the other two groups, with a slight female predominance [33]. The TTF-1 (−)/OTP (+) cluster describes patients with PC that are generally centrally located and exhibit an indolent biologic behavior, typically presenting as TCs, with low Ki-67, without an infiltrative type of growth or DIPNECH.

### 4.2. What We Do Not Know

Currently, the pathological characteristics of PC in AYA patients remain poorly defined. While a higher incidence of genetic syndromes, such as MEN-1, is presumed in this population compared to adults, the exact incidence of PC in AYA patients with MEN-1, as opposed to pediatric patients, remains unknown. Furthermore, the reasons for a diagnostic delay in the AYA population are still unclear. Given the findings in the study by Papaxoinis G et al. [33], it is likely that PCs in AYAs present with indolent biological behavior and a low proliferative index, features consistent with the nature of NETs within a MEN-1. However, the available literature is too heterogeneous and inconclusive to support any definitive conclusions. In terms of IHC, the scientific community is lacking data on the features in the AYA population that may be related to the clinical conditions of patients (smoking addiction, alcohol use, weight, etc.) and allow identifying some risk factors in this category.

## 5. Treatments and Survival

### 5.1. What We Know

Surgery with systematic nodal dissection remains the standard of care for localized PCs regardless of the patient’s age [1]. Endobronchial approaches, by contrast, are typically reserved for palliative purposes (due to the site of the PC, e.g., carina, or the patient’s performance status and comorbidities). A parenchymal-sparing resection is to be preferred over pneumonectomy, and lymphadenectomy should include at least six nodal stations [34]. This is particularly true given the notable incidence of lymph node metastases even in low-grade PCs (27% in TCs and 47% in ACs). Currently, no benefit has been reported for adjuvant treatment, which should, therefore, be considered and personalized case-by-case only in selected high-risk N2 AC patients.

In metastatic disease, systemic treatment may include hormone therapies, target therapies, and chemotherapy, or, within clinical trials, radioligand therapy (RLT), according to tumor biology and progression patterns (Table 1).

(1) Somatostatin analogues (SSAs). SSAs remain a cornerstone in the management of NETs, particularly for tumors expressing somatostatin receptors (SSTRs). Although their effects are primarily attributed to binding SSTRs—most commonly SSTR type 2—leading to inhibition of hormone secretion and potential antiproliferative consequences, some studies suggest indirect mechanisms of SSA action, such as paracrine/autocrine effects, antiangiogenic, or immunomodulatory properties [35,36]. In this setting, the clinical efficacy of SSAs remains less well-defined compared to gastro-entero-pancreatic NETs (GEP-NETs). While prospective data are limited, two phase II studies on this topic, the LUNA and RADIANT-2 trials, have reported promising findings in terms of progression-free survival (PFS) and, in selected cases, modest tumor shrinkage [37,38]. Notably, the RADIANT-2 and LUNA trials evaluated everolimus, a target therapy, combined with SSAs (octreotide and pasireotide, respectively). However, only the phase III SPINET trial was discontinued due to poor accrual, leaving the issue of SSA indication in PCs still unresolved (NCT02683941). As a result, current recommendations for SSA use in PCs are primarily based on retrospective analyses. Among these, a pivotal study by Sullivan et al. reported stable disease in 77% of the PC population with a median overall survival (OS) of 58.4 months [39]. Similarly, Lenotti et al. recently provided further support for SSA use in PC patients, including a subset of AYA cases [40].
  Despite these encouraging outcomes, SSA use for PCs remains a matter of debate in clinical practice, given the absence of phase III confirmation and that a subset of patients may lack SSTR expression, as indicated by negative somatostatin receptor imaging (SRI). Although the indirect effect of SSAs in NETs was well documented, its indication in this setting appears comparatively weaker.
(2) Target therapies. Everolimus, an m-TOR inhibitor, was the first agent approved for PCs, based on the positive RADIANT-4 trial results [41]. According to current guidelines [1,34], everolimus is recommended after SSA failure or in first-line therapy in case of ACs or aggressive behavior. Among the other targeted therapies, cabozantinib, a tyrosine kinase inhibitor (TKI), has shown promising activity in a phase II trial that included both gastro-entero-pancreatic (GEP) NETs and PCs, and its role is currently under investigation in the ongoing LOLA trial [42,43]. In Asia, but not in Western Countries, surufatinib has been approved for use in PCs, based on the SANET-ep phase III trial demonstrating PFS benefit in extra-pancreatic NETs, including PC [44]. Regorafenib, an anti-VEGFR, also demonstrated potential efficacy in PCs in a recent phase II trial by Perez K et al. [45], but its role is still under evaluation.
(3) Chemotherapy. Chemotherapy remains a key option for metastatic PC, particularly in cases with high tumor burden or aggressive features. Preferred regimens include a combination of alkylating agents (oxaliplatin, streptozotocin, or temozolomide) with fluoropyrimidines (5-Fluorouracil or capecitabine) [46,47], offering a well-manageable toxicity profile and a good clinical benefit rate [48].
(4) Radioligand therapy (RLT). At present, 177-Lu-DOTATATE (^177^Lu) or 90-Yttrium (^90^Y) RLT has shown promising results only in retrospective series involving PC patients [34,49]. The scientific community is waiting for the results of the phase III LEVEL trial, currently evaluating ^177^Lu RLT vs. everolimus in the PC population [50].
(5) Others. Radiotherapy or other interventional radiology techniques are typically reserved for a palliative intent [18].

In some cases, PC may be related to clinical syndromes due to an atypical production of peptide. Carcinoid Syndrome (CS) is the most common, primarily related to an increasing secretion of serotonin by the tumoral cells [34]. SSAs are the standard of care for the treatment of CS, but in some refractory cases, options may include interferon-alpha, RLT, or chemotherapy, or locoregional treatment, such as radiotherapy or embolization [51,52,53]. More rarely, ectopic Cushing’s syndrome (ECS) occurs in 2–6% of PC patients, due to an ACTH-independent production of cortisol [54]. Also in this case, management involves both antitumoral strategies (systemic and locoregional therapies) and cortisol synthesis inhibitors as ketoconazole, metyrapone, or osilodrostat [55,56].

Finally, the tumor-agnostic use of targeted therapy based on specific mutation patterns is emerging. Dramatic responses have been reported with alectinib in PCs harboring EML4-ALK fusion [57,58] and entrectinib in cases with NTRK mutation [59], highlighting the importance of comprehensive molecular profiling in advanced disease.

### 5.2. What We Do Not Know

When considering curative treatments for locoregional stages in AYA patients, case series occasionally report undertreatment with endobronchial approaches, even in patients who are otherwise eligible for surgery. The indication is likely influenced by the physician’s intention to avoid subjecting a young patient to lung surgery, as well as by the generally indolent biology of the disease compared to others. In the article by Luckraz L et al., pediatric, AYA, and adult populations were treated with an endobronchial approach for TCs. The authors reported a 10-year disease-free survival of 94% and a 10-year median overall survival of 84% with a median follow-up of 8.8 months, in line with other case series and higher compared to the 10-year survival rate for AC patients (31–67%) [54]. While these results may be relevant for patients over 75 years of age included in the analysis, they are arguably inadequate for AYA patients given their significantly longer life expectancy [60]. Prospective studies focusing specifically on the AYA population are therefore warranted to better define the appropriate surgical approach, evaluate disease-specific survival and mortality, and assess long-term outcomes related to the type of surgical intervention performed.

When addressing advanced or metastatic diseases, a significant gap in the current literature concerns the use of systemic therapies in the AYA population with NETs, including PC. Specifically, there is a notable lack of data derived from cohorts of AYA patients. In most of the published trials and retrospective analyses on systemic treatments, the AYA population was not included or analyzed as part of broader adult cohorts, without a dedicated subgroup analysis by age. For instance, the study by Lenotti et al., for example, is one of the few analyses that included patients under the age of 39 years. However, even in this case, the number and the characteristics of the AYA population were not separately reported.

The same issue is also evident in prospective studies on targeted therapies. For instance, both the LUNA and RADIANT-2 trials enrolled adult patients aged 18 years or over, including substantial numbers of patients under 65 years. Nevertheless, no disaggregated data have been made publicly available regarding how many AYA patients were included, nor provide detailed information on their clinical characteristics. Conversely, the RADIANT-4 trial did report enrollment of AYA patients; however, since it pooled GEP NETs with PCs, it is not possible to determine the exact number of AYA patients with PCs.

A retrospective analysis by Crona J et al. analyzed temozolomide use in the PC cohort, including AYA patients. The study reported a partial response in 14% of patients and stable disease in 52% but the AYA population was assessed within a broader adult case series, limiting the ability to draw definitive conclusions specific to this subgroup [60] (Table 1).

**Table 1:** Systemic treatments and availability data on AYA patients.

Therapy	Target	Available Data on AYA Population	
SSA	SSTRs	Lenotti et al. [40]	Available but not stratified by age
		LUNA [37]	Available but not stratified by age
		RADIANT-2 [38]	Available but not stratified by age
		RADIANT-4 [41]	No AYAs enrolled
Everolimus	mTOR	LUNA [37]	Available but not stratified by age
		RADIANT-2 [38]	Available but not stratified by age
		RADIANT-4 [41]	No AYAs enrolled
Cabozantinib	VEGFR	LOLA [42]	Ongoing
Surufatinib	VEGFR	SANET-ep [44]	Available but stratified among those < and ≥65 years old
Regorafenib	VEGFR	Perez K et al. [45]	Available but not stratified by age
Chemotherapy	NA	Granberg D. et al. [47]	Available on three AYA patients
		Taymeyah Al-Toubah et al. [48]	No AYAs enrolled
		Crona J. et al. [61]	Available but not stratified by age
Radioligand Therapy	SSTRs	ESMO guidelines [34]	Available but not stratified by age
		Malandrino L et al. [49]	Available but not stratified by age
		LEVEL trial [50]	Ongoing
Alectinib	EML4-ALK fusion	Lei X et al. [57]	No available AYA case report
		Liu N et al. [58]	No available AYA case report
Entrectinib	NTRK	Zhang W et al. [59]	No available AYA case report

SSAs: somatostatin analogues; SSTRs: somatostatin receptors; VEGFR: Vascular Endothelial Growth Factor Receptor; EML4-ALK: Echinoderm Microtubule-associated protein-Like 4—Anaplastic Lymphoma Kinase; NTRK: Neurotrophic Tyrosine Receptor Kinase.

In terms of palliative treatment, only one case report described the use of brachytherapy in choroidal metastasis in an 18-year-old adolescent with PC, who experienced glaucoma as an early adverse event. No data regarding outcomes after a further systemic progression of disease has been reported.

Therefore, in metastatic disease, systemic treatment recommendations for AYA patients with pulmonary carcinoids are currently extrapolated from adult data, leaving a critical gap in understanding the efficacy and early and late effects of these therapies within this younger population. Old studies about other diseases have shown that alkylating agents contribute to fertility impairment in approximately 60% of males, and radiotherapy below the diaphragm depressed fertility by 25% in both sexes [62]. In female patients, chemotherapy-induced premature ovarian insufficiency not only compromises fertility but also leads to significant bone density loss due to estrogen deficiency, a serious concern during this critical stage of development. From the time of diagnosis of a rare tumor in an AYA patient, the ensuing psychological and social impact can be profound, especially for individuals who are fully engaged in academic or professional settings with established routines or who are embarking on new family responsibilities.

Special attention should be given to MEN-1 cases, as oncologic diseases are not the only clinical concern in this population (Table 2). In a case series including both AYA and adult MEN-1 patients, a 10-year overall survival rate of 71% was reported, underlining that most deaths were attributed to causes unrelated to lung NETs [22]. On the other hand, within the same cohort, the prevalence of thymic NETs was 3.7%, with a disease-specific 10-year survival rate of just 25%, underscoring the particularly poor prognosis of these tumors in MEN-1 patients. Given the risk of thymic NETs, pituitary oncologic disease, pancreatic NETs, and further PCs, current guidelines recommend radiologic surveillance with an MRI or CT scan beginning as early as 10–15 years of age [63,64]. Therefore, this makes the impact of lifelong imaging particularly significant for AYA patients. Given these indications, the scientific community needs to first consider the long-term risks of radiation-induced malignancies associated with early and repeated imaging. A recent case series estimated a 0.49% risk of second cancers due to cumulative radiation effects in MEN-1 patients [65], suggesting an effort to further minimize the exposure. Beyond medical risks, the burden of continuous follow-up also has substantial implications for the quality of life, considering both the financial and psychological costs of bearing the expenses related to follow-up care. For a MEN-1 AYA patient with PC diagnosis engaged in school, work, or starting a family, the financial and psychological costs of ongoing surveillance can be considerable.

Managing follow-up in MEN1 patients requires a balance between ensuring timely tumor detection and minimizing the physical, emotional, and socioeconomic burden of surveillance.

As a scientific community, we currently lack sufficient data addressing these complex issues in the PC AYA population, highlighting the urgent need for focused research to better understand and address their unique psychological, social, and medical challenges.

## 6. Conclusions

Lung NETs remain a rare subset of thoracic neoplasms; however, PCs represent a significant proportion of primary pulmonary tumors in AYAs, a population often overlooked in clinical research. As observed across other oncologic diseases, despite the peculiar biological and clinical characteristics of AYA patients, this population remains significantly understudied. Consequently, current management strategies largely mirror adult or pediatric treatment paradigms, which may not fully address the unique clinical, molecular, and psychosocial factors faced by AYA individuals. According to the topic of our analysis, it becomes evident that there is a conspicuous absence of AYA-specific diagnostic and management frameworks for PCs; our review underscores several critical unmet needs, both in diagnostic and therapeutic fields. The limited literature dedicated to AYAs with a PC diagnosis highlights the urgent need for age-tailored investigative strategies and bespoke models of care.

Bringing the existing gap between data on PC management derived from adult or pediatric cohorts and the AYA population is crucial. The development of international collaborative efforts and designing prospective, AYA-focused clinical studies are imperative to deepen the understanding of this peculiar category of patients. Such efforts would not only improve clinical decision-making and promote the development of evidence-based, age-appropriate therapeutic strategies but also address broader issues such as survivorship, fertility preservation, and integration into educational or professional life. This need is particularly pressing for both subsets of AYA patients: those with localized and metastatic disease. In the former, the surgical management of a localized PC, requiring lobectomy or even pneumonectomy, carries a substantial risk of long-term respiratory morbidity, which can have profound implications for quality of life in this population. In the latter, chronic exposure to systemic therapies may result in a cumulative burden of iatrogenic toxicity, presenting unique challenges that further emphasize the need for individualized, age-sensitive management strategies.

To address these critical gaps, future research should prioritize the establishment of prospective, international registries specifically for AYA patients with a PC, with a focus on fertility-preserving interventions, long-term quality of life (QoL) outcomes, and psychosocial support measures. Developing robust, age-specific clinical trials and longitudinal studies will be essential to generate evidence-based guidelines tailored to the unique needs of this population, ultimately improving both survival and life quality for AYA patients with PCs.

## Figures and Tables

**Table 2:** MEN-1 follow-up issues in AYA patients.

MEN-1 Risks	Implication
Thymic cancer	Annually, an MRI or CT scan
Parathyroid adenoma	Annually, calcium, PTH
Insulinoma	Annually, glucose, fasting insulin
Pituitary Adenoma	Annually, PRL, IGF-1. Every 3 years MRI
Gastrinoma	Annually gastrin
Other NETs	Annually, MRI/CT scan for the specific district
Adrenal Adenoma	Annually, abdomen MRI/CT scan
Second cancers due to cumulative radiation effects	Considering specific follow-up

MRI: magnetic resonance imaging; CT: computer tomography; PTH: Parathyroid Hormone; PRL: prolactin; IGF-1: Insulin-like Growth Factor 1; NETs: neuroendocrine tumors.
